# Hypnotism: Its Value to the Dental Specialist

**Published:** 1896-02

**Authors:** George F. Grant

**Affiliations:** Boston, Mass.


### HYPNOTISM: ITS VALUE TO THE DENTAL SPECIALIST.[1]

BY DR. GEORGE F. GRANT, BOSTON, MASS.

It is fair to state at the outset of this paper that it is written more in view of eliciting discussion of the subject than of imparting any instruction as to the direct means of inducing hypnosis for the purpose of lessening the pain or reducing the dread of dental operations.

This subject teems with interest for us as any subject dealing with an agent for which such claim is made must necessarily possess. Every considerate operator would be made happy by the safe, sure abolition of pain from the operations he is called upon to perform. It is a safe proposition that up to the present time no such agent has been obtained.

I am at present considering hypnosis in this list, though I am willing to withdraw it upon conviction.

My experience in practice is like that of most others. A common question now from patients is, "What do you think of hypnotism?" In order to give a reasonably intelligent reply, one must read up and obtain a smattering, at least, of what has been written on the subject, with a special view to its applicability to his use and needs.

I believe that the conditions governing the application of this agent to medicine or surgery are so widely different from those with which we have to do, that very little except the general principles can be applied to dental practice.

It seems too great a trespass upon your time to enter into the history of hypnotism, for it extends very far back into the earliest of recorded history. My wish is to take up the latest writers and observers on the subject and glean from them what can be made

---

[1] Read before the American Academy of Dental Science, October 2, 1895.

valuable to us. One will find here rather puzzling variations of views.

First, as to the methods to be employed in the induction of the hypnotic state. Second, as to what percentage of people are susceptible to hypnotic suggestion. There are many other points upon which the opinions of students of this science differ, but I have selected those bearing most directly upon what it is desired to consider in a short essay.

The first great question is upon the means of producing hypnosis.

Abbé Faria said, in 1815, "The cause of sleep was in the person who was to be sent to sleep." "This (says Moll) is the main principle of hypnotism and of suggestion."

Besides this we have the rapidly-revolving mirror of Luys, used to produce speedy and extreme fatigue of the eye, the magnetic pass used by older magnetizers, the sound produced suddenly by striking a large gong, or a sudden ray from a Drummond light.

The effect can be produced through a sense of touch, even by gentle stroking of the skin. Others produce it by touches on the forehead, pressure upon the eyelids, etc. Then comes fixed attention, which is considered by some as the only means, while others combine it with mental methods, as Bernheim does.

Dr. Bonwill, in a paper on this subject, lays great stress on self-assertiveness on the part of the operator, and that "suggestion should be made in a loud, commanding tone, like a general commanding an army."

Dr. Warren and Dr. Faught, in discussing the paper, agreed that loudness of speech and commanding tone were quite unnecessary, as the same result could be obtained by a quiet tone.

Professor Newbold says, "Of all the means of heightening suggestibility with which we are acquainted, none is so easy of application and certain in its effects as the concentration of attention and limitation of the conscious field."

Dr. Osgood, of this city, says that the passes are unnecessary and smack of charlatanism.

We have here, then, many opinions as to the proper method of inducing this state.

Moll says, "Which of the above methods, or which combination of them, is the best for practical use? is a question the answer to which is not so simple that every one who has made a dozen experiments is justified in trying to reply to it."

I will now call your attention to the question of susceptibility. We here find a great difference in opinion between investigators.

Some claim the ability to hypnotize all subjects, even against their will (Donats), while others place the percentage very low.

Liébeault claims ninety-two per cent., Delbeouf eighty per cent., Bernheim and Ford claim over eighty per cent., while Bottey claims only thirty per cent., and Moll only twenty per cent. To compare, sift, and digest all these views seems no slight task for a man who is making the investigation with the purpose of adding one more agent to his list of aids in painless operation.

It seems to be generally agreed among men who have used hypnotic suggestion as a therapeutic agent, or as a means of performing painless surgical operations, that many complicated conditions exist, or may arise, which can only be properly met with by a systematic training, followed by careful and oft-repeated experiments. All this precedes the application of this science. We must first master the subject, then apply it to our specialty, if we intend making use of it.

Here a thought occurs which to me seems to have an important bearing upon our view of expediency.

The medical man would in all probability not find it necessary to induce the hypnotic state in many cases daily, nor would the surgeon perform operations continuously every day for from six to eight hours, as the dentist does. The majority of patients who visit the dentist require operations of a painful nature, while of those visiting a physician only a small percentage require or receive immediate surgical treatment.

This seems to me an important point for consideration, as even the most skilful or enthusiastic hypnotist would find that, whether he gave himself up to his subject or the subject submitted wholly to him, there would be so great a drain upon his mental force from such a continuous exertion, that when added to the physical and mental strain, inseparable from daily practice, it would tax the ordinary practitioner beyond his strength.

This seems a moderate view of this aspect of the question. While most of the men who have written, especially upon the dental side of the question, have given the impression that the art is easy of application, the very reverse is true of what the medical writers have to say. This is rather remarkable in view of the peculiarly sensitive organs upon which dental operations are to be performed; and, further, that all authors are not agreed that insensibility to pain is a feature of hypnosis. One rather gets the idea that it is a condition of unconsciousness of pre-existing pain, rather than insensibility to inflicted pain. A consideration of these observations rather leads to the hypothesis that the dentist sometimes mistakes a faith established by his reassuring manner for an hypnotic condition.

I think Dr. Bonwill's paper on hypnotism is a stronger exposition of what may be accomplished by considerate preparation of a patient for operation than it is of the value of hypnotic suggestion.

There is this much to be said of the results of investigation of this subject in the hospitals abroad, notably those of France and Germany, where most is known and the most extensive experiments have been made, that their results can only be regarded as establishing the existence of this great power for the benefit of mankind under conditions of nativity, habit of obedience, and strong predisposition to accept what is offered in the way of treatment. We all know that hospital practice is a widely different thing from office practice, and, if I am rightly informed, the physicians of foreign hospitals exercise far greater powers over the patients in such institutions than are permissible in kindred institutions in this country.

In order to restrict this paper to as close a bearing on the application of hypnotic suggestion in our specialty as could consistently be accomplished, I was obliged to omit much that is interesting,—namely, its psychological and physiological aspects, the dangers which of necessity are to be recognized and avoided; also the various theories advanced in explanation of the phenomena exhibited during hypnosis.

In my opinion the more thoroughly one reads and investigates the subject, the more clearly the fact is established that for our purpose it presents too many complex problems and requires much further investigation by minds trained to that especial kind of work. If it is to be used as an adjunct in dental operations, patients should bring their trained hypnotist to the dental chair, leaving the dental specialist to the performance of his legitimate functions.

In support of this last clause I cannot find better words than those of Dr. Osgood: "The dentist has no business to try to relieve every trouble of which a patient may complain, any more than I have to clear out a dental cavity without proper education and experience."

To that remark I give a hearty amen, and think it contains a whole world of application.

Upon the question as to whether a dental specialist might use hypnosis, after a course of training under an expert, I should say that weighing every consideration, those which I have tried to present, with those which lack of space forces me to omit, it is well to be (in the words of Professor Newbold) "sceptical as to facts and cautious as to theories."

